# Population Status of *Pan troglodytes verus* in Lagoas de Cufada Natural Park, Guinea-Bissau

**DOI:** 10.1371/journal.pone.0071527

**Published:** 2013-08-07

**Authors:** Joana S. Carvalho, Tiago A. Marques, Luis Vicente

**Affiliations:** 1 Centre for Environmental and Marine Studies, Department of Biology, Lisbon University, Campo Grande C2, Lisboa, Portugal; 2 Centre for Research into Ecological and Environmental Modelling, University of St. Andrews, St. Andrews, Scotland; 3 Centro de Estatística e Aplicações da Universidade de Lisboa, Lisboa, Portugal; SUNY College of Environmental Science and Forestry, United States of America

## Abstract

The western chimpanzee, *Pan troglodytes verus*, has been classified as Endangered on the IUCN Red List since 1988. Intensive agriculture, commercial plantations, logging, and mining have eliminated or degraded the habitats suitable for *P. t. verus* over a large part of its range. In this study we assessed the effect of land-use change on the population size and density of chimpanzees at Lagoas de Cufada Natural Park (LCNP), Guinea-Bissau. We further explored chimpanzee distribution in relation to landscape-level proxies of human disturbance. Nest count and distance-sampling methods were employed along 11 systematically placed linear transects in 2010 and 2011. Estimated nest decay rate was 293.9 days (%CV = 58.8). Based on this estimate of decay time and using the Standing-Crop Nest Count Method, we obtained a habitat-weighted average chimpanzee density estimate for 2011 of 0.22 nest building chimpanzees/km^2^ (95% CI 0.08–0.62), corresponding to 137 (95% CI 51.0–390.0) chimpanzees for LCNP. Human disturbance had a negative influence on chimpanzee distribution as nests were built farther away from human settlements, roads, and rivers than if they were randomly distributed, coinciding with the distribution of the remaining patches of dense canopy forest. We conclude that the continuous disappearance of suitable habitat (e.g. the replacement of LCNP's dense forests by monocultures of cashew plantations) may be compromising the future of one of the most threatened Guinean coastal chimpanzee populations. We discuss strategies to ensure long-term conservation in this important refuge for this chimpanzee subspecies at its westernmost margin of geographic distribution.

## Introduction

In the last decades, primate populations have suffered great demographic declines [1], [2]. These declines are due to several reasons, all having human activities and/or infectious disease epidemics as their core basis. However, little is known about how these threats translate into actual decrease in population size. Poaching, pet trade, slash-and-burn agriculture, deforestation associated with logging and agricultural activities, large-scale agricultural plantations, and other threats explain the biodiversity loss and fragmentation of several primate habitats worldwide [3], [4].

On the large scale distribution patterns of species are shaped by environmental and historical constraints [5], [6], [7]. On the small scale behavioural characteristics including territoriality, location of nesting sites, predation, and competition for food or mates determine where a species is found [8]. Today, human disturbance, quantifiable by population density, socio-economic and cultural factors, and the extent of roads and highways [9], [10], [11], is one of the major determinants of wildlife distributions [12], including chimpanzees [13], [14]. Primate distributions in Africa have been greatly affected by the expansion of road networks [15], [16], not only providing access to settlers but facilitating illegal hunting and logging [11], [17], [18]. In West and Central Africa, hunting is one of the greatest threats due to the dependence of local populations on bushmeat, for subsistence and for commerce [19], [20], [21]. Rivers can act as natural barriers shaping primate distribution patterns [7], [22], while at the same time allow for an easy transport of bushmeat [9].

Many studies have analyzed primate distributions with respect to different levels of human disturbance [19], [23], [24], [25], but few have provided a detailed quantification of the relationship [13], [14], [26], [27]. The impact of human activities on chimpanzee populations has been evaluated over large areas [14], [27], but there are few quantitative studies that have been conducted at a small geographic scale [11], [13], [21].

The Western chimpanzee *Pan troglodytes verus* has been listed as Endangered on the IUCN Red List since 1988 [28]. *Pan t. verus* has, nonetheless, undergone a considerable population reduction over the last 20 to 30 years [29]. Its range encompasses nine West African countries, although it is already considered rare or close to extinction in four of them: Burkina-Faso, Ghana, Guinea-Bissau, and Senegal [30]. Junker et al. [14] carried out a meta-analysis for eight taxa of African great apes that assessed continent-wide suitable environmental conditions and how they had changed over 20 years. They found that the western chimpanzee had suffered a decline of 11% in the area of suitable environmental conditions since 1992. The Cantanhez National Park in Guinea-Bissau has suffered the same loss of chimpanzee habitat (11%) since 1986 (surveys in 1986, 1994 and 2003; [13]).

**Figure 1:**
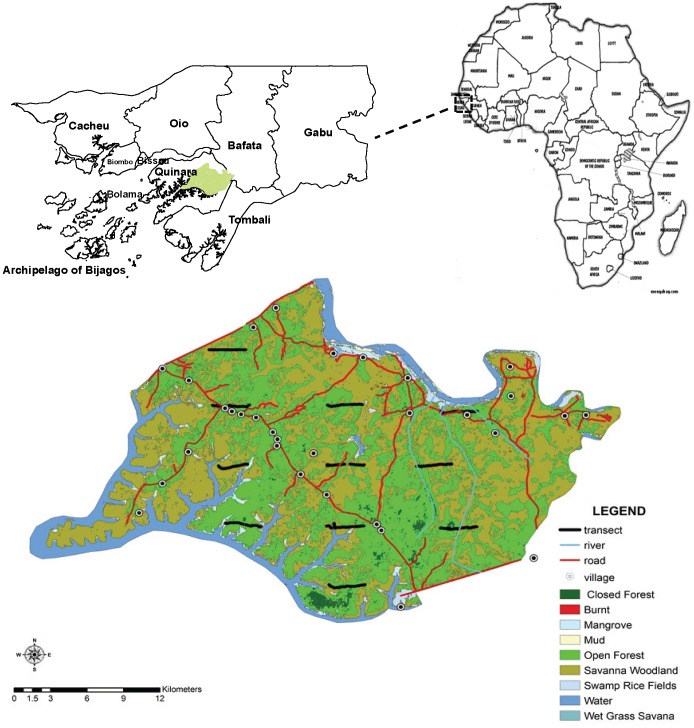
Location of the 11 linear transects inside the protected area Lagoas de Cufada Natural Park. The location of the Republic of Guinea-Bissau and respective administrative regions is shown.

Population estimates for this subspecies range from 21,300 to 55,600 individuals [30], with 600–1000 individuals in Guinea-Bissau [3]. Questionnaire surveys suggest that the range of chimpanzees is restricted by humans [31]. In Guinea-Bissau, the highest human population densities are found in the north of the country (http://www.bestcountryreports.com/Population_Map_Guinea-Bissau.php) and suitable habitat for *P. t. verus* is found only in the south [13]; in the south-west, in the region of Tombali, including the Cantanhez Forest and Cacine Basin, and in the region of Quinara, particularly in Lagoas de Cufada Natural Park (LCNP), and in the east in Boé (Figure 1) [32], [33].

The first report on the status of *P .t. verus* in this country dates from 1940 (Monard 1940 *in* [34]) and only recently have studies provided updated assessments [6], [35], [36], which, however, have focused only on a small area of the country or have employed a less robust study design [31], [33], [36] than is recommended [37]. For LCNP, the evidence available before 2008 suggests that chimpanzees occurred in the forests surrounding 23 villages, while in other areas they were scarce and seriously threatened by deforestation, poaching and increasing human populations, in others there were no signs at all of their presence [38]. No reliable estimates of the current population size and density of *P.t verus* in this important protected area were available.

In this paper, we provide robust density and population size estimates for the western chimpanzee population in LCNP using a distance-sampling approach [37], [39]. We also assess patterns of chimpanzee occurrence inside LCNP in relation to landscape-scale covariates of human disturbance, such as roads, rivers, and settlements.

## Methods

### Ethics Statement

All research was conducted under permissions from *Instituto da Biodiversidade e Áreas Protegidas* (IBAP), Guinea-Bissau. No animals were captured or handled during this study.

### Study Site

Guinea-Bissau is a small (36,125 km^2^) West African country (Figure 1) with relatively flat topography [3]. Patches of primary forest remain in the north-west and south-west of the country, in the regions of Cacheu, Quinara, and Tombali [3]. Landscape satellite images have shown, however, that dense canopy forests continue to decline in extent and number, being replaced by open canopy forests and savannah-woodland [40], [41] (Table S1). The dense canopy forests are being replaced by subsistence farming of rice, sugarcane and maize, and cashew plantations [40].

LCNP is located in the region of Quinara, in southern Guinea-Bissau, between 11°34′ and 11°51′ N and 14°49′ and 15°16′ W (Figure 1) [42], [43]. The climate is characterized by an average annual temperature of 26°C and an average annual rainfall of 2200 mm, with a pronounced rainy season from June to October/November [44]. LCNP is an internationally recognized Ramsar site and covers an area of 890 km^2^ [33]. It is managed by a governmental organization, the *Instituto da Biodiversidade e das Áreas Protegidas* (IBAP). IBAP faces severe funding and personnel limitations despite multiple threats to the park's integrity. Different ethnic groups (around 11,000 people) live in the park, relying extensively on natural resources for their survival. The villages are close to roads or water sources (rivers or lagoons). The villagers' livelihoods depend on subsistence farming, and bushmeat hunting is common.

### Chimpanzee Nest Surveys

As chimpanzees in LCNP are not habituated to humans and, as such, very elusive (only 10 encounters were recorded in 2011), we relied on nest surveys for estimating population densities [17], [23], [45], [46]. Nest counts are a useful surrogate for estimating ape densities and monitoring their populations over time [47], [48]. Most studies recommend using line transect surveys [15], [18], [49], [50], during which all the nests visible from the transect line are counted either individually [17], [45] or in groups [23], or both [46]. Two nest count techniques are recommended: Standing-Crop Nest Counts (SCNC) and Marked Nest Counts (MNC). SCNC consists of only a single visit to all transects, counting all nests irrespective of their age class, whereas MNC consists of counting only nests built between successive visits to the same transect, with all nests removed in the first visit, within an interval short enough to guarantee that no new nests will disappear between repeated visits. SCNC is logistically easier [15], [23], [50], [51], although it requires independent estimates of rates of nest production and nest decay. MNC, on the other hand, is the only alternative when nest decay rate is lacking [17], [18], [46], [51]. The pros and cons of these methods are well described in the literature [18], [47].

Here, we used a hybrid approach, depending on survey year, sampling unit, and nest count method. We estimated (1) densities of chimpanzee nests for 2010 and 2011 using line transect surveys and SCNC, (2) chimpanzee density for 2010 using strip transect surveys and MNC, (3) nest decay rate in 2010 for LCNP, and (4) subsequently, based on line transect sampling and SCNC, the chimpanzee density for 2011 using our estimate of nest decay rate and published information on nest production rate.

Following the recommendations for an adequate study design [37], [39], 11 linear transects (each one 3 km long) were superimposed over LCNP as a grid of equally-spaced (5×6 km) parallel lines (Figure 1). Our design adhered to the assumptions underlying distance sampling [37].

All field work was conducted by JC, during 10 months in the dry seasons of 2010 and 2011. Habitat types along each line transect were classified based on canopy coverage as either dense canopy forest, open canopy forest, or savannah-woodland [42], [44] and geo-referenced to calculate the exact proportion of each in these sampling units. Line transects were visited four and five times, respectively, in 2010 and 2011, at biweekly intervals. The transects were walked at a steady speed of about 1 km/h [46], [51].

Only data on individual nests were collected, considering that nest groups were hard to identify as nests showed the highest aggregation in dense canopy forests. Whenever a nest was found, the perpendicular distance from the transect line to the nest was measured with a tape measure or range finder (Bushnell Yardage Pro Sport 450). To avoid double counting, nest trees were tagged with a rope. Nests observed during the 2011 survey were geo-referenced using a Global Positioning System (GPS Garmin 60) (only one reading was taken when there were several nests close to each other in the same tree).

## Analyses

### Distance Sampling Nest Surveys

We used the software DISTANCE 6.0 [52] to estimate nest encounter rate, the nest detection function, and the densities of nests and chimpanzees. We first explored the distance data in histograms, considering different cut-off points and fitting a half-normal model without adjustment terms to get a first feel for the shape of the detection function and to assess the best truncation distance (*w*). Some data truncation is recommended to avoid problems fitting the tail of the distribution, and 5% has been recommended as a plausible omnibus value for *w* [37]. Subsequently, we considered a range of other models implemented in DISTANCE to assess which model provided the best fit to the data. The best model was selected using Akaike's Information Criterion (AIC), and by evaluating the goodness of fit of the models based on the standard chi-square, the Kolmogorov-Smirnov and the Cramér-von Mises tests [37].

### Estimation of Nest Densities from Line Transects using SCNC

Only nest data from the first visit to any particular transect in each year were used to obtain the nest detection function by habitat. We opted not to include data from subsequent visits for nest density estimation because, because even under the assumption that during biweekly intervals no newly built nest will disappear, old nests were detected during repeated visits [46].

Nest data were examined following the procedures described above for model selection and model evaluation. First, we estimated nest densities for each habitat. Nest density 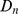
was estimated using the conventional distance sampling estimator as 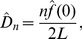
(1)where *n* represents the number of the detected nests, 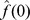
is the estimated probability density function of detected nests evaluated at distance 0 and *L* is the total length of transects [37]. Global nest density was obtained as a weighted average of habitat specific estimates, with weights given by habitat area.

### Estimation of Chimpanzee Density and Nest Decay Rate from Strip Transects using MNC

The linear transects were also regarded as constituting a grid of randomly positioned strips. Unlike line transect sampling, standard strip transect sampling assumes that all objects (either individuals or indirect evidence of their occurrence) within a distance 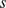
along transects are detected, and providing a large enough sample size an unbiased estimator of density and precise estimates of abundance can be obtained [15], [53], [54]. To maximize the likelihood of detecting all nests within distance *s*, we used the 2010 dataset considering only nests from the second visit onwards to find the distance *s* for which we could consider that all new nests were detected, i.e., that would allow us to define sensible strip transects. The width of strip transects (*s*) was defined by the distance over which the shoulder of the detection function extended. We considered habitat-specific strip transects, given that the width over which it is reasonable to assume that all nests are detected was expected to be habitat dependent (compare also [55]).

Following data exploration as described above, we estimated chimpanzee density by habitat, and then global density weighted by habitat, as 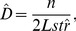
(2)where *n* represents the number of new nests detected within the strip transect from the second visit onwards, *L* is the total strip transect length, *s* is the width of the strip transect (taken from the shoulder of the detection function as described above), *t* is the number of days elapsed between the first and last survey, and 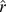
is the daily nest production rate [37]. As an estimate of nest production rate for our study area or Guinea-Bissau is lacking, we used a published estimate of 1.143 nests built per animal per day (%CV = 3.51) from Taï National Park, Ivory Coast [56]. 95% confidence intervals for nest encounter rates and density estimates were calculated in R version 2.15.3 [57] using a nonparametric bootstrap procedure (999 resamples).

Using the above 2010 estimates of nest density and chimpanzee density, we subsequently obtained an estimate of nest decay rate by rearranging the following equation and solving it for 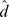


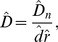
(3)where 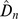
denotes the estimate of nest density, 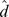
is the nest decay rate (days) and 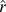
is the nest production rate per day [37]. Nest decay rate was calculated for 2010.

The variance for the decay rate estimator can be approximated via the delta method [58] as 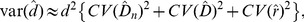
(4)where *CV* represents the coefficient of variation of the corresponding estimate, i.e., the standard error of the estimate divided by the estimate.

### Estimation of Chimpanzee Densities from Line Transects using SCNC

Based on the estimated rate of nest decay and again using the estimate of the daily rate of nest production from Taï National Park, we were able to apply the SCNC technique [59] to estimate a habitat-weighted average of chimpanzee densities using equation 3 for the 2011 data.

### Relationship between Nest Distribution and Landscape-Scale Covariates

Nests were used as an indirect measure of the presence of chimpanzees [13]. A randomization test was performed using the package *COIN* in R version 2.15.3 [57] to assess relationships between the spatial distribution of chimpanzee nests and a set of landscape-scale variables which can be regarded as proxies for human disturbance: principal rivers, roads (including main and secondary roads), and human settlements. To determine whether nests were distributed in a non-random fashion with respect to these variables we compared the mean distances between nest locations and each environmental feature to mean distances generated in the same way based on random locations of 214 (number of independent nest locations in the data) nests within transects. This procedure was repeated 1000 times and statistical significance was assessed by recording the number of times the mean value from random locations was lower than the observed value for nest locations [60]. We used the Geographic Information System (GIS) ARCMAP 9.3 package to calculate the shortest straight-line distance between each nest and a given environmental feature. All spatial layers were projected into Universe Transverse Mercator (UTM) Zone 28N. Environmental digital data were made available through the CARBOVEG project (http://carboveg-gb.dpp.pt/) or taken from Amaro [61]. To ensure that sampling of random points (*n* = 214) fell within the area surveyed, a buffer was constructed along both sides of the transects and limited by the maximum distance at which a nest was observed from the transect line surveys (84 m). In addition, to investigate the distribution of habitats in relation to the environmental features considered, we also plotted the measured distances grouped by habitat type and tested for statistical differences.

## Results

### Chimpanzee Nest Surveys

Survey effort for SCNC was 67.2 km, whereas 235.2 km were walked for MNC. Line transects were composed mostly of savannah-woodland (46.81%), followed by dense canopy forest (26.28%), open canopy forest (9.97%), agricultural areas (10.08%), herbaceous savannah (5.35%), rivers or lagoons (1.23%) and human settlements (0.28%) (Figure S1). These relative proportions of habitat types in LCNP constitute a good representation of their occurrence countrywide (Table S1). In 2010, 211 nests were detected, 182 of them during the first visit. A total of 248 nests were recorded in 2011, 117 of those during the first visit. The highest count during the first visit is the natural consequence of nest accumulation over time.

Once corrected for estimated habitat specific detectability (see results below) nests were found mostly in dense forest (71.55 %), and fewer nests were recorded in the two habitats with less canopy coverage: savannah-woodland (17.98%) and open forest (10.48 %). Distances at which nests were detected from the line transect differed significantly among habitat types (Kruskal-Wallis test, 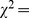
40.82, *df* = 2, *p*<0.001) (Figure S2), being greatest in savannah and shortest in dense forest.

### Estimation of Nest Densities from Line Transects using SCNC

Truncating the data at 42 meters, a uniform model with a cosine adjustment provided the best fit for the 2010 dataset (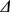
AIC = 0.77; the reported values of 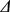
AIC correspond to the comparison with the second best model unless otherwise noted). Open forests showed the highest nest encounter rate followed by dense forests and savannahs (Table 1). Habitat-specific nest density estimates were substantially higher for open and dense canopy forests compared to savannahs (Table 1). For 2010, the global nest density estimate for LCNP was 167.97 nests per km^2^ (95% CI 55.61–507.34).

**Table 1:** Comparison of nest count, nest encounter rate (nests/km) and nest density estimates (nests/km^2^) between 2010 and 2011 of the chimpanzees in Lagoas de Cufada Natural Park.

Year	Survey habitat	No. of nests	Nests/km [95% CI]^a^	Nests/km^2^ [95% CI]^a^	% CV^b^
2010	Global^c^			167.97 [55.61–507.34]	44.21
	DF	65*	7.37 [1.77–30.64]	229.68 [55.22–955.30]	66.17
	OF	67*	20.03 [4.88–82.26]	364.37 [90.07–1,474.10]	54.36
	SAV	36*	2.29 [0.81–6.44]	27.28 [9.70–76.72]	48.20
2011	Global^c^			75.56 [27.21–209.86]	42.22
	DF	72*	8.16 [3.36–19.81]	233.21 [96.02–566.39]	38.87
	OF	17*	5.08 [1.01–25.59]	129.79 [26.39–638.39]	63.52
	SAV	13*	0.83 [0.19–3.52]	11.82 [2.77–50.36]	71.23

a CI, confidence interval.

b Coefficient of variation.

c Average nest density weighted by habitat.

*Distance truncated at 42 m (2010) and 35 m (2011).

DF- dense canopy forests; OF- open canopy forests; SAV- savannah-woodlands.

Applying a truncation distance of 35 m, a uniform model with a cosine adjustment (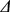
AIC = 3.06) best fitted the 2011 data. The number of nests observed in dense forests was similar to 2010, whereas far fewer nests were observed in open forests and savannahs, leading to much lower estimates of nest encounter rate for these habitats compared to the previous year (Table 1). Nest density was highest in dense forests, followed by open forests and savannahs, resulting in a global nest density estimate of 75.56 nests per km^2^ (95% CI 27.21–209.86) estimated for the entire park, less than half of the previous year's estimate (Table 1). Note, however, the considerable overlap in the 95% CIs, suggesting that this reduction is not statistically significant.

### Estimation of Chimpanzee Density and Nest Decay Rate from Strip Transects using MNC

Based on AIC, a half-normal model with cosine adjustment showed the best fit to the 2010 data after truncation at 44 m (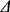
AIC = 2.08). Based on the detection function, strip transects of different width were obtained for each habitat (Table S2). Habitat-specific density estimates were similar in dense and open forests (Table S2). The park-wide weighted average estimate was 0.50 nest builders per km^2^ (95% CI 0.18–1.39), corresponding to about 311 chimpanzees for LCNP. Using these estimates of chimpanzee densities and the nest densities estimated above for 2010 (Table 1), nest decay rate was estimated to be 293.9 days (%CV = 58.80).

### Estimation of Chimpanzee Densities from Line Transects using SCNC

**Figure 2:**
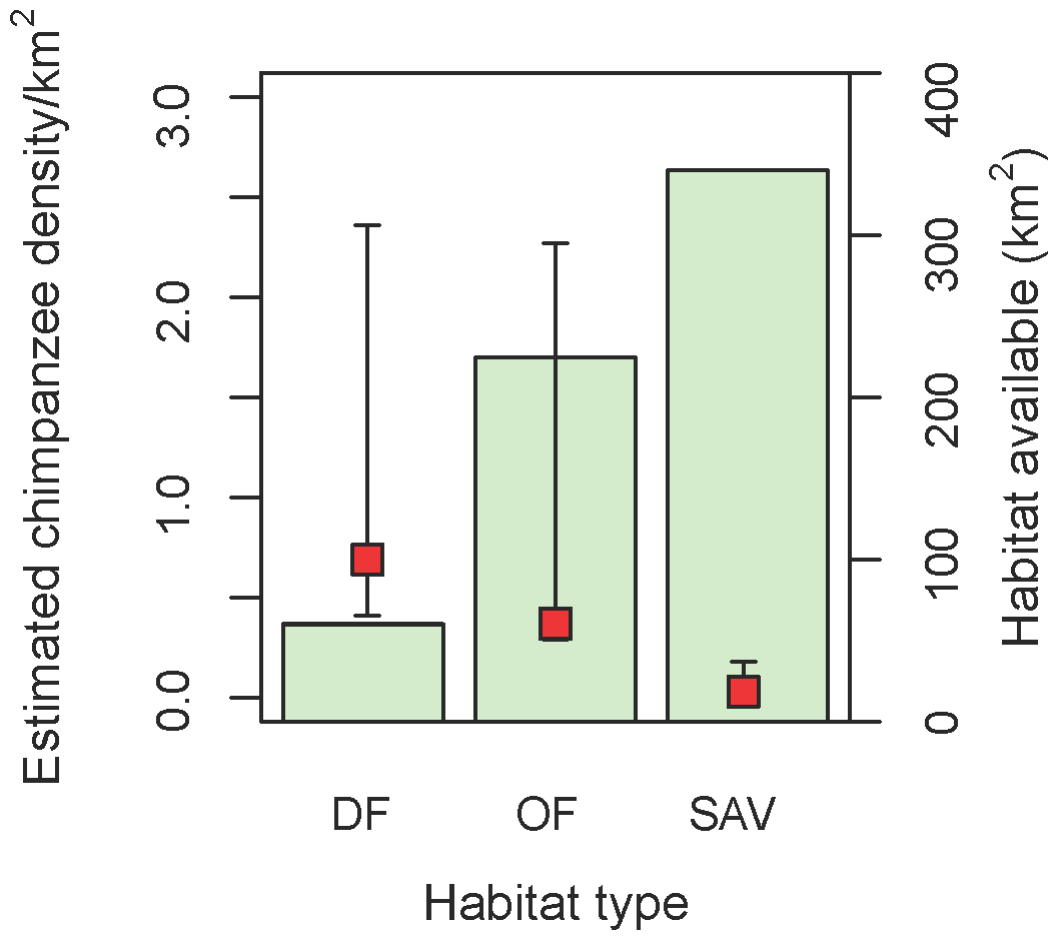
Estimates of chimpanzee density by habitat type for 2011. Estimates were based on line transect surveys, applying the Standing-Crop Nest Counts method. Also indicated is the area occupied by each type of habitat in Lagoas de Cufada Natural Park. Dense canopy forests (DF), open canopy forests (OF) and savannah-woodlands (Sav) were the habitats considered.

Using the above estimated rate of nest decay, chimpanzee density estimates for 2011 were highest in dense forests and lower in savannahs (Figure 2, Table 2). The habitat-weighted average for LCNP was 0.22 nest builders per km^2^ (95% CI 0.08–0.62), corresponding to 137 chimpanzees.

**Table 2:** Chimpanzee density estimates (builders/km^2^) for each habitat and for the Lagoas de Cufada Natural Park obtained in 2011 based on Standing-Crop Nest Counts, using our estimated rate of nest decay.

Survey habitat	Density (builders/km^2^)	95% CI^a^ (builders/km^2^)	%CV^b^
Global^c^	0.22*	0.08–0.62	42.22
Dense canopy forests	0.69*	0.28–1.67	38.87
Open canopy forests	0.37*	0.08–1.90	64.00
Savannah-woodlands	0.03*	0.01–0.15	71.23

a Confidence interval

b Coefficient of variation.

c Average nest density weighted by habitat.

*results using the nest decay rate of 293.9 days (%CV = 58.80).

It is important to note that the greatest chimpanzee density was estimated for the least available habitat type (Figure 2).

### Relationship between Nest Distribution and Landscape-Scale Covariates

**Figure 3:**
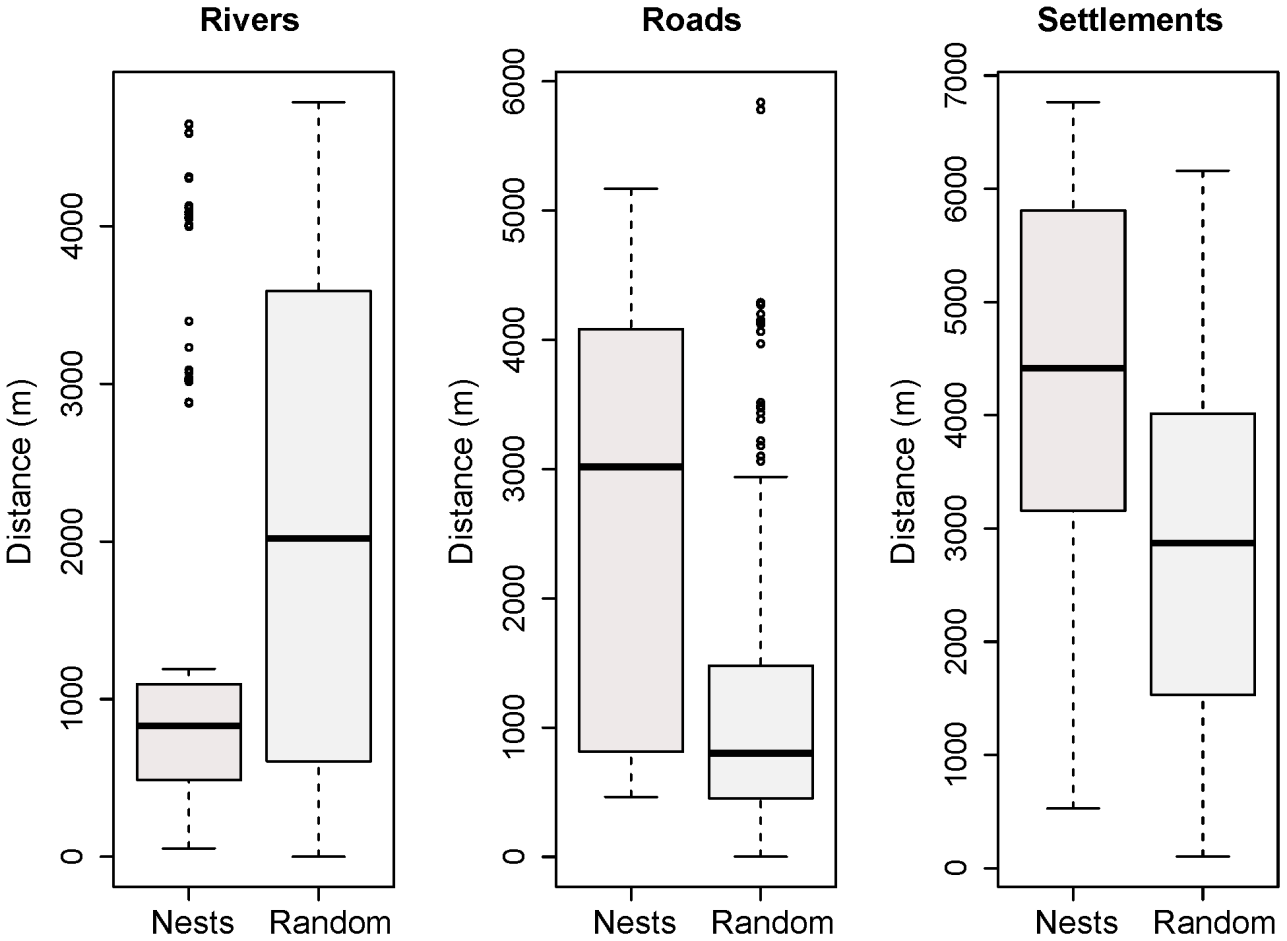
Nearest distances from chimpanzee nests and random points to the landscape-scale covariates. Rivers, roads, and human settlements were considered as proxies of human disturbance.

The randomization test showed that the spatial distribution of chimpanzee nests differs significantly from a random pattern for all three environmental variables examined. Chimpanzees prefer to build their nests farther away from roads (*Z* = 9.55, *p*<0.001), settlements (*Z* = 7.60, *p*<0.001), and rivers (*Z* = −5.81, *p*<0.001) than would be expected by chance. On average nests were observed farther away from settlements (4.13 km, 95% CI 3.88–4.37), than from roads (2.58 km, 95% CI 2.37–2.80) or rivers (1.28 km, 95% CI 1.10–1.45) (Figure 3).

**Figure 4:**
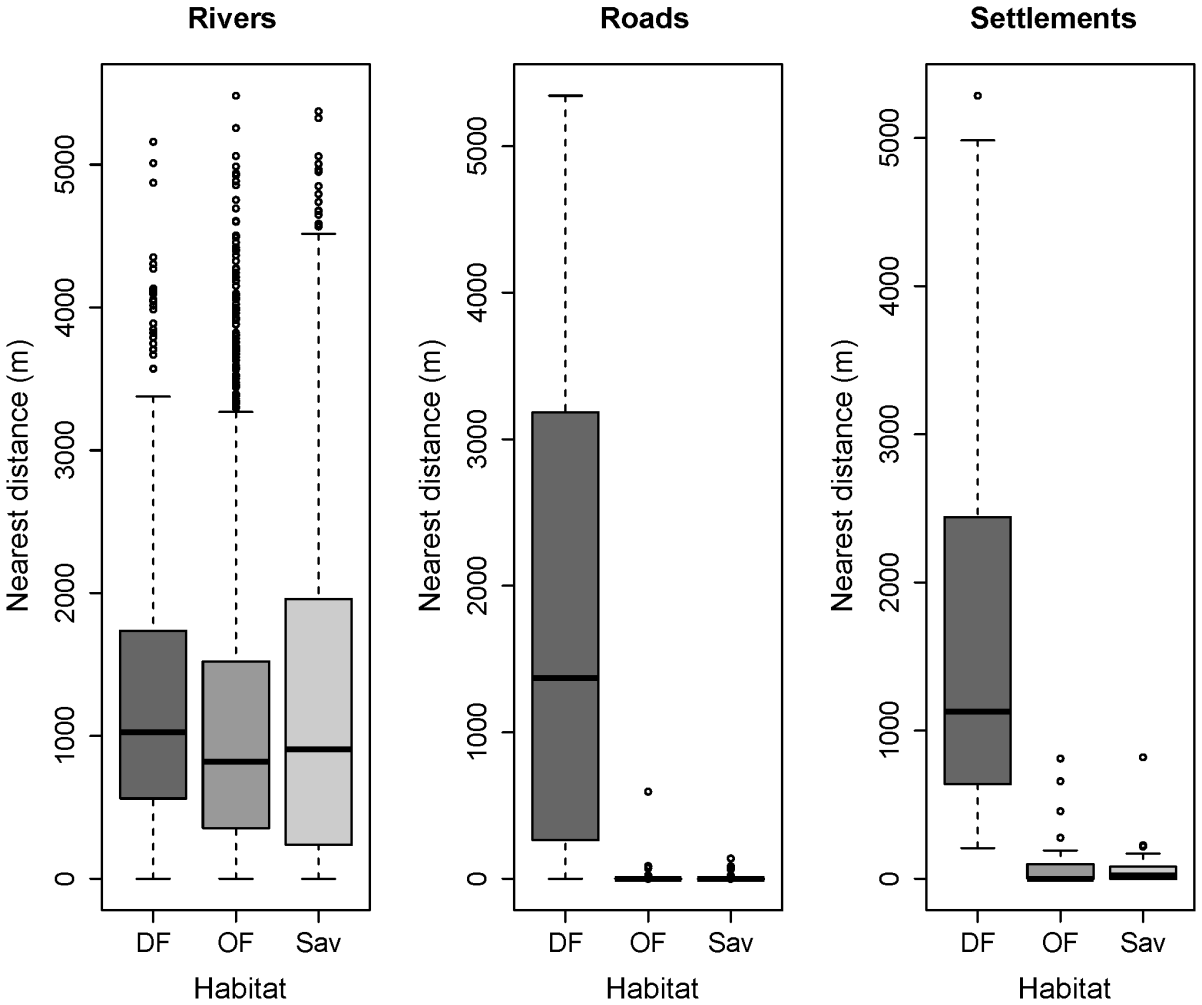
Nearest distances from habitats used by chimpanzees for nest building to the landscape-scale covariates. Rivers, roads, and human settlements were considered as proxies of human disturbance. Dense canopy forests (DF) are located farther from all landscape variables, and shortest distances were obtained for habitats with a lower tree canopy cover, such as open canopy forests (OF) and savannah-woodlands (Sav).

The distribution of habitats differed significantly in relation to the environmental features (Kruskal-Wallis test: rivers 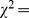
10.55, *df* = 2, *p*<0.05; roads 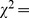
124.29, *df* = 2, *p*<0.001; settlements 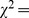
56.89, *df* = 2, *p*<0.001), whereby habitats with a lower tree canopy cover (open forests and savannahs) were found closer to all landscape variables, contrasting with the large distances obtained for dense forests (Figure 4).

## Discussion

### Chimpanzee Population Density and Size

We presented chimpanzee density estimates at LCNP for 2010 (0.22 nest builders per km^2^, 95% CI 0.08–0.62) and 2011 (0.50 nest builders per km^2^, 95% CI 0.18–1.39). While it is true that the 95% confidence intervals overlap considerably, hence not suggesting a large population change, we believe the apparent doubling of the point estimates is an artefact (i.e. a consequence of the large CV's associated with these estimates) and these numbers provide nonetheless a good comparison for future studies in this region. By comparison with previous studies for Guinea-Bissau we report the lowest chimpanzee density estimate (Table S3). There are three published studies for this country that provide density estimates based on distance sampling, however, just one of them employed a random sampling of line transects [62] and the remainder used abandoned trails or trails used by locals for access to crops or for hunting [36], [63].

Our estimates of chimpanzee densities for each habitat type show an inverse relationship with habitat availability (Figure 2), which highlights the preference for building nests and the suitable nesting conditions offered by dense canopy forests. The present results confirm previous studies [36], [62], [63] in demonstrating that tree canopy cover plays an important role in habitat choice for nest building in chimpanzees from Guinea-Bissau, in contrast to what has been found at other sites [23], [64], [65], [66]. As an adaptation when dealing with declines of their preferred habitat, chimpanzees evidently opt to nest in savannah-woodlands [23], [67]. LCNP chimpanzees also use open canopy forests for nest building, which taken together underscores the importance of considering all habitat types for estimating chimpanzee densities, and also with respect to conservation efforts.

Method selection is a compromise between sound and well-established methodologies and the available resources and personnel [68]. Population size estimates of primates rely on certain assumptions, which vary depending on the different methodologies available [47], [68], [69], [70]. Several studies applied nest count methods (Table S3). Nest production rates are usually taken from long-term monitoring of habituated chimpanzees due to the difficulty of observing wild chimpanzees [47], [59]. Despite the differences found in this variable between sites and seasons, many studies used non-site specific information (see Table S3).

Our estimate of nest decay corresponds only to the dry season and with 293.9 days (%CV = 58.80) for 2010 was close to those reported from other locations within the western chimpanzee's range (Table S4). Further studies are required during the rainy season to compare robust estimates of the life span of nests from LCNP with those from other sites. Although we applied SCNC to estimate chimpanzee densities among habitats the overlap of the confidence intervals indicates low power to detect changes (Figure 2, Table 2). In future research we suggest that the decay rate presented here be incorporated when using SCNC. However, as decay rate depends on unmeasured covariates that may vary temporally and spatially [56], [71], to avoid bias and obtain an accurate population size estimate, we recommend a new estimate under actual survey conditions [72] by monitoring the decay of new nests during successive visits.

Other techniques have emerged that deal with the time consuming process of monitoring the decay of a large and diverse sample of nests to obtain accurate estimates of life span of nests [73]. For example, assuming a Markov chain for the state of a nest, with an absorbing state which represents nest disappearance, van Schaik et al. (1995) were able to estimate the time a nest takes to disappear based on the observation of nests (and their corresponding state) over time. For more details see [47], [71], [74]. Current work in progress based on our data set uses both state space models (D. L. Borchers, pers. comm.) and hidden Markov Models (R. Langrock, pers. comm.) to simultaneously estimate nest decay rate and abundance.

### Chimpanzee Distribution in Relation to Human Disturbance

Even though chimpanzees reportedly show a certain ability to coexist with humans [75], places they consider safe for nest building have previously been shown to be distant from human settlements, roads, and rivers [11], [21], [27], further pointing towards a perhaps not surprising negative influence of human disturbance on chimpanzee distribution.

A recent meta-analysis by Junker et al. (2012) showed that measures of human impact such as proximity to settlements make a large contribution to the loss of suitable ecological conditions for chimpanzees. Historically most of the chimpanzee populations in Guinea-Bissau had human settlements within their range, and hence people regularly come into contact with chimpanzees on roads (main and secondary roads), in cultivated areas, and around the edges of forest fragments [76].

Roads have been shown to be prejudicial for chimpanzee populations as they facilitate poaching and illegal hunting, and indirectly boost illegal logging [17], [18], as has also been reported for other primates and other taxa such as ungulates, rodents and carnivores [9], [10].

Cashew nuts are Guinea-Bissau's principal cash crop, representing 90% of the country's exports since 2000 [16], [77]. Most of the roads and settlements in LCNP are surrounded by extensive cashew plantations. Replacement of native forest by these monocultures reduces the availability of those trees that have canopies suitable for chimpanzees to build their nests. The cashew pulp is widely appreciated by many taxa, and some farmers reported that chimpanzees sometimes split branches while trying to reach the fruit at greater heights, leading to irreversible damages of trees and often resulting in chimpanzee-human conflict (S. Camará, pers. comm.). This study coincided with the period of cashew harvesting (March to late June), when the number of people inside the park, as well as road traffic, usually increases. Unlike the park residents, in general, these temporary harvest workers show little awareness with respect to the conservation of park biodiversity, compromising and undermining the conservation efforts by guards and residents.

The distributions of several forest-dwelling primate taxa in west and central Africa have been shown to be limited by rivers; larger rivers have a greater barrier effect on species distribution of forest taxa than smaller rivers, as observed for the Congo River and the rivers bounding the Dahomey Gap [7]. The Dahomey Gap, a dry savanna corridor interrupting the West African rainforest, has been a barrier for primate species either by its aridity or by its flanking rivers, the Volta and Niger [7]. The main rivers surrounding Lake Tumba, Congo, have also acted as a barrier, influencing the distribution of bonobos and chimpanzees [22]. LCNP is delimited by two main rivers, the Corubal in the north and the Buba River in the south, which limits the chimpanzee distribution north and south of the protected area (Figure 1). People living in remote areas of LCNP with limited road access use navigable rivers as transportation routes, which could have the same negative impact on chimpanzees as roads.

### Methodological Implications

Chimpanzee populations worldwide are declining at alarming rates and an immediate reclassification of chimpanzees to a status of “critically endangered” has been recommended (e.g. [1]). In light of such declines there is an urgent need to standardise appropriate designs and methodologies for long-term monitoring if the conservation of remaining chimpanzee populations is a priority for biodiversity management [78]. In this context, it is essential to consider the bias associated with a certain survey methodology, as well as its efficiency and cost-effectiveness [54]. How can we reliably detect population declines within and between protected areas? What is the best way to provide baseline information for long-term population monitoring? In this regard it is crucial to stress that using trails or reconnaissance surveys might result in biased density estimates, compared to line transect surveys based on randomly placed transects, which, although more labour-intensive and expensive, should be the method of choice as they provide unbiased and potentially more accurate population estimates [37]. SCNC have been a viable and economical way to detect population declines, and procedures of monitoring programs and assessment of human impacts are performed using MNC surveys. The Ape Populations, Environments, and Surveys (A.P.E.S) Database aims to compile existing great ape survey data and make density and distribution data accessible to the scientific community (http://apes.eva.mpg.de ). Our data will be made available in this database to help incentivize more standardized monitoring efforts and enable comparisons between different study sites [2], [14].

### Final Considerations

Long-term population monitoring in LCNP, an important refuge for coastal populations of the western chimpanzee, would be highly desirable and may be achieved by investing in local training and capacity building. In general, human communities need to be included in conservation management, for instance by employing local people as park guards or tourist guides, to ensure effective long-term conservation [30]. As a mitigation measure to minimize human-chimpanzee conflict it would be desirable to concentrate crops, including future cashew plantations, in zones that are already disturbed and where environmentally sustainable practices could be implemented [11]. We also recommend an effective control of illegal hunting by strengthening and enforcing the existing law, which forbids poaching (Decree No. 21/1980).

As ours, several other studies have shown the importance of protected areas for the preservation of stable primate populations. As there is evidence, however, that primates continue to use resources outside protected areas, recent studies advocate a landscape-scale conservation approach that takes into account the ecological requirements of species at larger spatial scales [27], [79], [80].

Finally, our study contributes to our understanding of ecological patterns and how chimpanzees are influenced by human disturbance. In this regard it is, however, important to keep in mind that the chimpanzee-human relationship is complex, and present-day distribution patterns may not be explained alone by currently measurable variables as they may in part also reflect species adaptive responses to historical human activities.

## Supporting Information

Figure S1 **Relative proportions of habitat type found along each line transect.**
(TIFF)

Figure S2 **Boxplots showing the distances at which nests were detected from the line transects.** The data from 2010 and 2011 surveys were combined, and distances truncated at >60 m.
(TIF)

Table S1 Deforestation rate in Guinea-Bissau based on Landsat satellite imagery from 1990 to 2007 [data from Oom et al. [40]].
(DOCX)

Table S2 Chimpanzee density estimates (builders/km^2^) for each habitat and for the *Lagoas de Cufada* Natural Park obtained in 2010 based on marked-nest counts, using strip transect surveys.
(DOCX)

Table S3 Estimates of chimpanzee densities (chimpanzees/km^2^) and population size reported for several study sites based on *nest count methods*. Estimates of chimpanzee densities from Guinea-Bissau are shown in italics.
(DOCX)

Table S4 Life span of nests from several study sites, including our estimate of nest decay for *Lagoas de Cufada* Natural Park.
(DOCX)
