# Supplementary figures and images for: Population Status of Pan troglodytes verus in Lagoas de Cufada Natural Park, Guinea-Bissau
Source: PLoS One. 2013 Aug 7;8(8):e71527. doi: 10.1371/journal.pone.0071527 (PMC3737107; doi:10.1371/journal.pone.0071527)

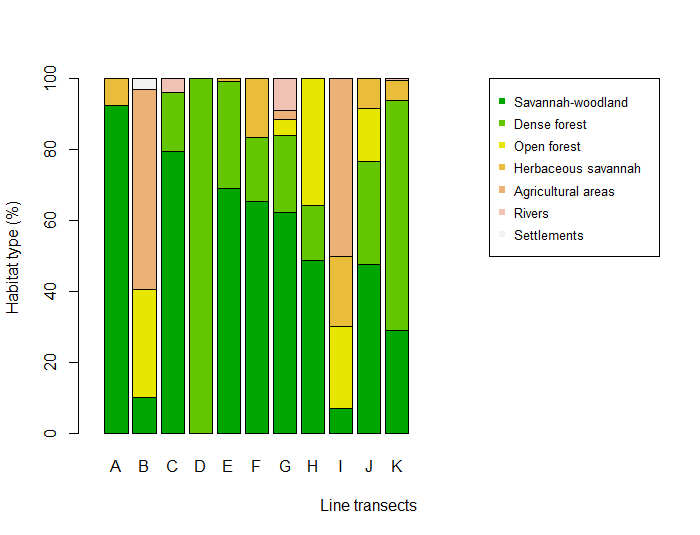

Supplement: Figure S1 — Relative proportions of habitat type found along each line transect. (TIFF) [file pone.0071527.s001.tiff]
