# Supplementary material for: Population Status of Pan troglodytes verus in Lagoas de Cufada Natural Park, Guinea-Bissau
Source: PLoS One. 2013 Aug 7;8(8):e71527. doi: 10.1371/journal.pone.0071527 (PMC3737107; doi:10.1371/journal.pone.0071527)
Supplement: Table S1 — Deforestation rate in Guinea-Bissau based on Landsat satellite imagery from 1990 to 2007 [data from Oom et al. [40]]. (DOCX) [file pone.0071527.s003.docx]

**Table S1**

| Habitat type | % per year | % of total area | % of area |
| --- | --- | --- | --- |
| Savannah-woodland | ↑ 0.76 | ↑ 13 | 48 |
| Mangroves | ↑ 0.83 | ↑ 14 | 9 |
| Open canopy forest | ↓ 1.17 | ↓ 15 | 25 |
| Dense canopy forest |  | ↓ 50 | 3 |
| Other | - | - | 15 |
