# Supplementary material for: Population Status of Pan troglodytes verus in Lagoas de Cufada Natural Park, Guinea-Bissau
Source: PLoS One. 2013 Aug 7;8(8):e71527. doi: 10.1371/journal.pone.0071527 (PMC3737107; doi:10.1371/journal.pone.0071527)
Supplement: Table S2 — Chimpanzee density estimates (builders/km2) for each habitat and for the Lagoas de Cufada Natural Park obtained in 2010 based on marked-nest counts, using strip transect surveys. (DOCX) [file pone.0071527.s004.docx]

**Table S2**

| Survey habitat | Density (builders/km^2^) | 95% CI^a^ (builders/km^2^) | %CV^b^ |
| --- | --- | --- | --- |
| Global^c^ | 0.50^*^ | 0.18-1.39 | 52.90 |
| Dense canopy forests | 1.10*^*^* | 0.00-2.42 | 72.21 |
| Open canopy forests | 1.09*^*^* | 0.00-3.62 | 62.11 |
| Savannah-woodlands | 0.00 | - | - |

^a^Confidence interval.

^b^Coefficient of variation.

^c^Average nest density weighted by habitat.

^*^after defining strip transects (DF, *s*=6m; OF, *s*=16m; Sav, *s* =35m).
