# Supplementary material for: Population Status of Pan troglodytes verus in Lagoas de Cufada Natural Park, Guinea-Bissau
Source: PLoS One. 2013 Aug 7;8(8):e71527. doi: 10.1371/journal.pone.0071527 (PMC3737107; doi:10.1371/journal.pone.0071527)
Supplement: Table S3 — Estimates of chimpanzee densities (chimpanzees/km2) and population size reported for several study sites based on nest count methods. Estimates of chimpanzee densities from Guinea-Bissau are shown in italics. (DOCX) [file pone.0071527.s005.docx]

**Table S3**

| Subspecies | Location | Study site | Estimates of density | Population size | Data source |
| --- | --- | --- | --- | --- | --- |
| ***P.t. verus*** | Guinea-Bissau | Cantanhez NP | *1.94-2.34* | 33-40 | Sousa [[1](#_ENREF_1)] |
|  |  | Gadamel | *0.90* | 33 | Sousa [[2](#_ENREF_2)] |
|  |  | Lagoas de Cufada Natural Park | *0.75 (0.27-1.67)* | 281 | Sousa [[3](#_ENREF_3)] |
|  |  |  | *0.22 (0.08-0.62)* | 137 | This study (2011 survey) |
|  | Guinea | Nationwide | N/A | 1,420-6,625* | Sugiyama and Soumah [[4](#_ENREF_4)] |
|  |  | Haut Niger NP | 0.87 | N/A | Fleury-Brugiere and Brugiere [[5](#_ENREF_5)] |
|  |  | Koulako | 1.09 | N/A | Ham, 1998 *in* Ganas [[6](#_ENREF_6)] |
|  |  | Moyeria | 0.90 | N/A | Ham, 1998 *in* Ganas [[6](#_ENREF_6)] |
|  |  | Siria | 0.26 | N/A | Ham, 1998 *in* Ganas [[6](#_ENREF_6)] |
|  |  | Diecke Forest | 0.17 | N/A | Ham, 1998 *in* Ganas [[6](#_ENREF_6)] |
|  | Ivory Coast | Taϊ NP | 0.09-1.7 | 11,676±1,168 | Marchesi et al. [[7](#_ENREF_7)] |
|  |  |  | 2.19 (core area) | 105 | Kouakou et al. [[8](#_ENREF_8)] |
|  |  |  | 0.15 (periphery) | 7 | Kouakou et al. [[8](#_ENREF_8)] |
|  | Liberia | Sapo Forest | 0.24 (0.18-0.77) | 240 | Anderson et al. [[9](#_ENREF_9)] |
|  | Mali | Bafing Forest Reserve | 0.27 | N/A | Pavy 1993 *in* Fleury-Brugiere and Brugiere [[5](#_ENREF_5)] |
|  |  | Faragama | 0.30 | N/A | Granier and Martinez 2004 *in* Fleury-Brugiere and Brugiere [[5](#_ENREF_5)] |
|  |  | Djakoli | 0.39 | N/A | Granier and Martinez 2004 *in* Fleury-Brugiere and Brugiere [[5](#_ENREF_5)] |
|  | Senegal | Niokolo Koba NP (Mt. Assirik) | 0.13 | N/A | Pruetz et al. [[10](#_ENREF_10)] |
|  |  | Fongoli | 0.09 | N/A | Pruetz et al. [[10](#_ENREF_10)] |
|  |  | Kharakhena | 0.08-0.19 | 34 | Boyer [[11](#_ENREF_11)] |
|  |  | Koudekouru (Bofeto) | 0.04-0.09 | 34 | Boyer [[11](#_ENREF_11)] |
|  | Sierra Leone | Gola Forest Reserves | 0.27 (0.18-0.41) | 305 (203-458) | Ganas [[6](#_ENREF_6)] |
| ***P.t. troglodytes*** | Camaroon | Dja Reserve | 0.79 (0.60-1.04) | N/A | Williamson and Usango, 1996 i*n* Morgan et al. [[12](#_ENREF_12)] |
|  | Central Africa | Nationwide | 0.01-0.13 | N/A | Carroll 1986 *in*  Blom et al. [[13](#_ENREF_13)] |
|  |  | Dzanga-Ndoki NP | 0.16 | 79 | Blom et al. [[13](#_ENREF_13)] |
|  | Congo | Lac Télé Community Reserve | 0.70 (0.4-1.3) | N/A | Poulsen and Clark [[14](#_ENREF_14)] |
|  |  | Goualougo Triangle | 1.53 (1.21-1.93) |  | Morgan et al. [[12](#_ENREF_12)] |
|  | Equatorial Guinea | Rio Muni | 0.31-1.53 | N/A | Jones and Sabater Pi, 1971 *in* Morgan et al. [[12](#_ENREF_12)] |
|  | Gabon | Belinga | 0.32 (0.03-0.49) | 64,173±13,000 | Tutin and Fernandez [[15](#_ENREF_15)] |
|  |  | Petit Loango Reserve | 0.78 | N/A | Furuichi et al. [[16](#_ENREF_16)] |
| ***P.t. schweinfurthi*** | Congo | Kahuzi-Biega | 0.40 | 7,670 (4,180-10,830) | Hall et al. [[17](#_ENREF_17)] |
|  |  | Kasese | 0.11 | 3,350 (1,420-5,950) | Hall et al. [[17](#_ENREF_17)] |
|  |  | Odzala NP | 2.20 | N/A | Bermejo [[18](#_ENREF_18)] |
|  | Tanzania | Malagarasi River to Karema | 0.21* | N/A | Kano, 1972 *in* Hashimoto [[19](#_ENREF_19)] |
|  |  | Kwitanga Forest | 0.69 (0.31-1.54) | 15 (7-34) | Ndimuligo [[20](#_ENREF_20)] |
|  | Uganda | Kalinzu Forest | 2.8 - 4.7 | 384-644 | Hashimoto [[19](#_ENREF_19)] |
|  |  | Kibale Forest | 1.97 | 1509 | Ghiglieri [[21](#_ENREF_21)] |
|  |  | Budongo Forest | 1.3 - 2.5 | 570-1066 | Plumptre and Reynolds [[22](#_ENREF_22)] |

*results based on questionnaires

NP- National Park

N/A- Not Available

**References for Table S3**

1. Sousa JV (2007) Densidade de Pan troglodytes verus e Veículos de Sensibilização Ambiental: Quatro Florestas de Cantanhez, República da Guiné-Bissau. Lisboa: Faculdade de Ciências da Universidade de Lisboa. 130 p.

2. Sousa FM (2009) Densidade de *Pan troglodytes verus* e uso de Recursos Naturais pela População Local, (Gadamael, República da Guiné-Bissau). Lisbon: Lisbon University, Sciences Faculty. 81 p.

3. Sousa J (2008) Densidade de *Pan troglodytes verus* (Chimpanzé da África Ocidental) no Parque Natural das Lagoas de Cufada, República da Guiné-Bissau. Lisbon: Faculdade de Ciências Sociais e Humanas, Universidade Nova de Lisboa. 55 p.

4. Sugiyama Y, Soumah A (1988) Preliminary survey of the distribution and population of chimpanzees in the Republic of Guinea. Primates 29: 569-574.

5. Fleury-Brugiere MC, Brugiere D (2010) High population density of *Pan troglodytes verus* in the Haut Niger National Park, Republic of Guinea: implications for local and regional conservation. International Journal of Primatology 31: 383-392.

6. Ganas J (2009) Population status survey and monitoring of western chimpanzee (*Pan troglodytes verus*) in the Gola Forest Reserve, Sierra Leone. Report to U.S. Fish and Wildlife Service.

7. Marchesi P, Marchesi N, Fruth B, Boesch C (1995) Census and distribution of chimpanzees in Cote D'Ivoire. Primates 36: 591-607.

8. Kouakou CY, Boesch C, Kuehl H (2009) Estimating Chimpanzee Population Size with Nest Counts: Validating Methods in Taı¨ National Park. American Journal of Primatology 71: 447-457.

9. Anderson J, Williamson E, Carter J (1983) Chimpanzees of Sapo Forest, Liberia: Density, nests, tools and meat-eating. Primates 24: 594-601.

10. Pruetz JD, Marchant LF, Arno J, McGrew WC (2002) Survey of savanna chimpanzees (*Pan troglodytes verus*) in Southeastern Sénégal. American Journal of Primatology 58: 35-43.

11. Boyer KM (2011) Chimpanzee Conservation in Light of Impeding Iron Ore Mining Project in SE Senegal. Ames, Iowa: Iowa State University. 68 p.

12. Morgan D, Sanz C, Onononga J-R, Strindberg S (2006) Ape abundance and habitat use in the Goualougo Triangle, Republic of Congo. International Journal of Primatology 27: 147-179.

13. Blom A, Almaši A, Heitkönig IMA, Kpanou JB, Prins HHT (2001) A survey of the apes in the Dzanga-Ndoki National Park, Central African Republic: a comparison between the census and survey methods of estimating the gorilla (*Gorilla gorilla gorilla*) and chimpanzee (*Pan troglodytes*) nest group density. African Journal of Ecology 39: 98-105.

14. Poulsen J, Clark C (2004) Densities, distributions, and seasonal movements of gorillas and chimpanzees in Swamp Forest in Northern Congo. International Journal of Primatology 25: 285-306.

15. Tutin CEG, Fernandez M (1984) Nationwide census of gorilla (*Gorilla g. gorilla*) and chimpanzee (*Pan t. troglodytes*) populations in Gabon. American Journal of Primatology 6: 313-336.

16. Furuichi T, Inagaki H, Angoue-Ovono S (1997) Population density of chimpanzees and gorillas in the Petit Loango Reserve, Gabon: Employing a new method to distinguish between nests of the two species. International Journal of Primatology 18: 1029-1046.

17. Hall JS, White LJT, Inogwabini B-I, Omari I, Morland HS, et al. (1998) Survey of Grauer's gorillas (*Gorilla gorilla graueri*) and eastern chimpanzees *(Pan troglodytes schweinfurthi*) in the Kahuzi-Biega National Park lowland sector and adjacent forest in eastern Democratic Republic of Congo. International Journal of Primatology 19: 207-235.

18. Bermejo M (1999) Status and conservation of primates in Odzala National Park, Republic of the Congo. Oryx 33: 324-332.

19. Hashimoto C (1995) Population census of the chimpanzees in the Kalinzu Forest, Uganda: comparison between methods with nest counts. Primates 36: 477-488.

20. Ndimuligo SA (2007) Assessment of Chimpanzee (*Pan troglodytes*) Population and Habitat in Kwitanga Forest, Western Tanzania. Johannesburg, South Africa: University of Witwatersrand. 66 p.

21. Ghiglieri MP (1984) The chimpanzees of Kibale Forest. New York: Columbia University Press. 226 p.

22. Plumptre AJ, Reynolds JF (1996) Censusing chimpanzees in the Budongo Forest, Uganda. International Journal of Primatology 17: 85-99.
