# Supplementary material for: Population Status of Pan troglodytes verus in Lagoas de Cufada Natural Park, Guinea-Bissau
Source: PLoS One. 2013 Aug 7;8(8):e71527. doi: 10.1371/journal.pone.0071527 (PMC3737107; doi:10.1371/journal.pone.0071527)
Supplement: Table S4 — Life span of nests from several study sites, including our estimate of nest decay for Lagoas de Cufada Natural Park. (DOCX) [file pone.0071527.s006.docx]

**Table S4**

| Subspecies | Location | Study site | Mean nest life span (days) | Season | Data source |
| --- | --- | --- | --- | --- | --- |
| ***P.t. verus*** | Guinea-Bissau | *Lagoas de Cufada* Natural Park | 293.9 | dry | This study (2010 survey) |
|  | Guinea | Haut Niger | 221.0 | both | Sugiyama and Soumah [[1](#_ENREF_1)] |
|  |  |  | 194.0 | both | Fleury-Brugiere and Brugiere [[2](#_ENREF_2)] |
|  | Ivory Coast | Taϊ NP | 91.2 | both | Kouakou et al. [[3](#_ENREF_3)] |
|  |  |  | 73.3 | both | Marchesi et al. [[4](#_ENREF_4)] |
| ***P.t. troglodytes*** | Gabon | Belinga | 113.6 | both | Tutin and Fernandez [[5](#_ENREF_5)] |
|  |  | Northeastern Gabon | 112.8 | N/A | Tutin and Fernandez 1981 *in* Anderson et al. [[6](#_ENREF_6)] |
|  | Central Africa | Dzanga-Ndoki NP | 50.9 | both | Blom et al. [[7](#_ENREF_7)] |
|  | Congo | Goualougo Triangle | 90.0 or 91.5? | both | Morgan et al. [[8](#_ENREF_8)] |
|  | Gabon | Lope | 106.0 | both | White, unpublished *in* Hall et al. [[9](#_ENREF_9)] |
| ***P.t. schweinfurthi*** | Tanzania | Mahale Mountains NP | 131.0 | both | Ihobe [[10](#_ENREF_10)] |
|  |  | Gombe NP | 36.0 | N/A | Moyer et al. [[11](#_ENREF_11)] |
|  |  | Ugalla Forest | 97.0 | N/A | Moyer et al. [[11](#_ENREF_11)] |
|  | Uganda | Budongo Forest | 37.2 | dry | Plumptre and Reynolds [[12](#_ENREF_12)] |
|  |  |  | 54.6 | Wet | Plumptre and Reynolds [[12](#_ENREF_12)] |
|  |  |  | 45.9 | both | Plumptre and Reynolds [[12](#_ENREF_12)] |
|  |  | Kibale | 111.0 | N/A | Ghiglieri [[13](#_ENREF_13)] |

NP- National Park

N/A- Not Available

**References for Table S4**

1. Sugiyama Y, Soumah A (1988) Preliminary survey of the distribution and population of chimpanzees in the Republic of Guinea. Primates 29: 569-574.

2. Fleury-Brugiere MC, Brugiere D (2010) High population density of *Pan troglodytes verus* in the Haut Niger National Park, Republic of Guinea: implications for local and regional conservation. International Journal of Primatology 31: 383-392.

3. Kouakou CY, Boesch C, Kuehl H (2009) Estimating chimpanzee population size with nest counts: validating methods in Taï National Park. American Journal of Primatology 71: 447-457.

4. Marchesi P, Marchesi N, Fruth B, Boesch C (1995) Census and distribution of chimpanzees in Cote D'Ivoire. Primates 36: 591-607.

5. Tutin CEG, Fernandez M (1984) Nationwide census of gorilla (*Gorilla g. gorilla*) and chimpanzee (*Pan t. troglodytes*) populations in Gabon. American Journal of Primatology 6: 313-336.

6. Anderson J, Williamson E, Carter J (1983) Chimpanzees of Sapo Forest, Liberia: Density, nests, tools and meat-eating. Primates 24: 594-601.

7. Blom A, Almaši A, Heitkönig IMA, Kpanou JB, Prins HHT (2001) A survey of the apes in the Dzanga-Ndoki National Park, Central African Republic: a comparison between the census and survey methods of estimating the gorilla (*Gorilla gorilla gorilla*) and chimpanzee (*Pan troglodytes*) nest group density. African Journal of Ecology 39: 98-105.

8. Morgan D, Sanz C, Onononga J-R, Strindberg S (2006) Ape abundance and habitat use in the Goualougo Triangle, Republic of Congo. International Journal of Primatology 27: 147-179.

9. Hall JS, White LJT, Inogwabini B-I, Omari I, Morland HS, et al. (1998) Survey of Grauer's gorillas (*Gorilla gorilla graueri*) and eastern chimpanzees *(Pan troglodytes schweinfurthi*) in the Kahuzi-Biega National Park lowland sector and adjacent forest in eastern Democratic Republic of Congo. International Journal of Primatology 19: 207-235.

10. Ihobe H (2005) Life span of chimpanzee beds at the Mahale Mountains National Park, Tanzania. Pan African News 12: 10-12.

11. Moyer D, Plumptre AJ, Pintea L, Hernandez-Aguilar A, Moore J, et al. (2006) Surveys of chimpanzees and other biodiversity in Western Tanzania. A Technical Report. 35 p.

12. Plumptre AJ, Reynolds JF (1996) Censusing chimpanzees in the Budongo Forest, Uganda. International Journal of Primatology 17: 85-99.

13. Ghiglieri MP (1979) Socioecology of chimpanzees in Kibale Forest, Uganda. Davis: University of California.
